# Nematode-bacteria interactions in bovine parasitic otitis

**DOI:** 10.1590/S1984-29612024081

**Published:** 2024-11-27

**Authors:** Makoto Enoki Caracciolo, Erika Verissimo Villela, Leandro dos Santos Machado, Maria Lúcia Barreto, Ana Cláudia de Paula Rosa, Eduardo José Lopes-Torres

**Affiliations:** 1 Laboratório de Helmintologia Romero Lascasas Porto, Departamento de Microbiologia, Imunologia e Parasitologia, Universidade do Estado do Rio de Janeiro – UERJ, Rio de Janeiro, RJ, Brasil; 2 Centro Multiusuário para Análise de Fenômenos Biomédicos, Universidade do Estado do Amazonas – UEA, Manaus, AM, Brasil; 3 Departamento de Microbiologia, Imunologia e Parasitologia. Faculdade de Ciências Médicas, Universidade do Estado do Rio de Janeiro – UERJ, Rio de Janeiro, RJ, Brasil; 4 Departamento de Saúde Coletiva Veterinária e Saúde Pública, Universidade Federal Fluminense – UFF, Niterói, RJ, Brasil; 5 Núcleo de Animais de Laboratório, Universidade Federal Fluminense – UFF, Niterói, RJ, Brasil; 6 Laboratório Multiusuário de Parasitologia, Departamento de Microbiologia, Imunologia e Parasitologia, Universidade do Estado do Rio de Janeiro – UERJ, Rio de Janeiro, RJ, Brasil

**Keywords:** Helminths, Metarhabditis, treatment, multiresistance, pathogenicity, microbiota, Helmintos, Metarhabditis, tratamento, multirresitência, patogenicidade, microbiota

## Abstract

Bovine parasitic otitis poses challenges in diagnosis, treatment and involves various agents, such as bacteria, fungi, mites, and nematodes. This study focused on the nematodes and bacteria isolated from the auditory canals of dairy cattle. A total of twenty samples were collected from dairy cattle in two states of Brazil. The results showed that *Metarhabditis freitasi* and *M*. *costai* nematodes were identified in 75% of samples. Bacterial species from the ear, identified via mass spectrometry, revealed that different strains were present in 65% of the cattle. *Mycoplasma* spp. were identified in 45% of samples through molecular techniques. Gram-negative bacteria and *Mycoplasma* spp. were exclusively found in nematode-infected cattle. Furthermore, the bacteria exhibited resistance to multiple antimicrobial classes, and demonstrating multiresistance. Electron microscopy revealed biofilm aggregates on the cuticle of *Metarhabditis* spp., suggesting a potential role of these nematodes in bacterial migration and interaction with nervous tissue. Thirteen bacterial strains demonstrated biofilm formation ability, indicating their potential pathogenic role. This research highlights the persistent and complex nature of parasitic otitis, emphasizing the significant role of nematode-bacteria associations in its pathogenicity. The presence of resistant strains and biofilm formation underscores the challenges in managing the diagnosis and treatment of bovine parasitic otitis.

## Resumo

A otite parasitária bovina apresenta desafios no diagnóstico e tratamento, envolvendo agentes como bactérias, fungos, ácaros e nematoides. Este estudo focou nos nematoides e bactérias de canais auditivos de gado leiteiro. Um total de vinte amostras foi coletado de bovinos em dois estados do Brasil. Os resultados mostraram nematoides *Metarhabditis freitasi* e *M*. *costai* identificados em 75% das amostras. Espécies bacterianas foram identificadas por espectrometria de massa, revelando a presença de diferentes cepas em 65% do gado. *Mycoplasma* spp. foram identificados em 45% das amostras usando técnicas moleculares. Gram-negativas e *Mycoplasma* spp., foram encontradas exclusivamente em bovinos parasitados. Além disso, bactérias exibiram resistência a várias classes de antimicrobianos e demonstraram multirresistência. A microscopia eletrônica revelou agregados de biofilme sobre a cutícula de *Metarhabditis* spp., sugerindo um possível papel desses nematoides na migração bacteriana e na interação com tecido nervoso. Além disso, treze cepas bacterianas demonstraram capacidade de formação de biofilme, indicando seu potencial patogênico. Este trabalho destaca a natureza persistente e complexa da otite parasitária, enfatizando o papel da associação entre nematoides e bactérias em sua patogenicidade. A presença de cepas resistentes a antimicrobianos e a formação de biofilme, ressalta desafios no manejo do diagnóstico e tratamento da otite parasitária.

## Introduction

Nematodes include a wide range of parasites with major socioeconomic importance (Ziliotto et al., 2024). The act of parasitizing cattle decreases the health of the animals through loss of body weight, reduced milk production, impaired ability to give birth to healthy calves, and high treatment costs in herds (Rashid et al., 2019). As a result, economic losses run into the billions of dollars due to the significant impacts of parasitism on production and the high costs of treatment in herds (Grisi et al., 2014; Strydom et al., 2023). Dairy cattle are commonly parasitized by gastrointestinal nematodes and other infectious agents, affecting productivity and potentially causing mastitis, lameness, fasciolosis, bovine viral diarrhea, dictyocaulosis, paratuberculosis, and infectious bovine rhinotracheitis (Charlier et al., 2009).

Bovine parasitic otitis can result from mite infestations and infection by nematodes of the genus *Metarhabditis* (Martins, 1985; Veloso et al., 2022). This phenomenon is particularly prevalent in dairy cattle breeds located in tropical and subtropical regions (Leite et al., 2012). Additionally, nervous system disorders may manifest, presenting symptoms such as head rotation, increased apathy, lack of coordination, salivation, and difficulties in chewing, including food accumulation in the animal's mouth (Duarte & Hamdan, 2004). The absence of a correct diagnosis and effective treatment leads to worsening of the condition, resulting in secondary bacterial infection. This results in a sudden decline on milk production and, in severe cases, the death of cattle (Leite et al., 2012). Additionally, *Rhabditis* nematodes, which are from the family of *Metarhabditis* species, have been reported in the human outer ear canal in Germany (Teschner et al., 2014).

There are limited data on the complex associations between *Metarhabditis* nematodes and bacteria in bovine otitis. In addition, the impacts of parasitism and the presence of bacterial species in the animal ear increase the risk of other bovine diseases (Sobral et al., 2020). The clinical diagnosis of parasitic otitis in cattle is mainly based on signs identified by farmers and laboratory diagnosis is rare and without a standardized protocol and without specific guidelines (Leite et al., 2012). Knowing the etiology of infections is crucial for selecting more effective treatments, minimizing the risk of disease spread, and preventing treatment resistance (Stracy et al., 2022). The likelihood of developing resistant strains is increased by the widespread use of antimicrobials, not only for treating animal diseases but also as a prophylactic measure and growth promoter, thereby impacting both animal and public health (Van Boeckel et al., 2017). Various bacterial genera and species, such as *Staphylococcus* spp., *Escherichia coli*, other enterobacteria, and bacteria from the Pasteurellaceae family, exhibit a multidrug-resistant profile (Freitas et al., 2018).

Different species of nematodes are isolated from soil and are directly associated with bacteria-feeding behavior or other interactions with these microorganisms (Donhauser et al., 2023; Liu et al., 2017). Biofilm formation can affect free-living nematodes, but details of this impact on parasite species are lacking. The biofilm of *Yersinia* spp. can block the mouth of *Caenorhabditis elegans*, impeding bacterial consumption and leading to the death of the nematode due to starvation (Atkinson et al., 2011). The biofilms formed by *Pseudomonas aeruginosa* and *Salmonella* can kill *C. elegans* after being internalized in the intestine (Tan et al., 1999; Desai et al., 2019), and *P. aeruginosa* can retard the motility of *C. elegans* via the production of a specific exopolysaccharide (Chan et al., 2021). Furthermore, bacterial pathogenicity increases significantly with the ability to form biofilms, which also contributes to antimicrobial resistance (Israel et al., 2018).

The association between bacteria and nematodes has been previously described in the context of bacterial translocation in intestinal nematodes (Castañeda et al., 2022). This process causes mucosal rupture, allowing these microorganisms to access tissue layers that are normally not colonized, thereby increasing inflammatory infiltration (Schachter et al., 2020). Information regarding the bacterial species and pathophysiological mechanisms associated with parasitic otitis is scarce, impeding infection control programs (Duarte & Hamdan, 2004). The associations between *Metarhabditis* and pathogenic bacteria remain unknown. In the present study, we propose a detailed protocol for isolating nematodes and bacteria from the ear of an animal. We report an increase in pathogenic bacteria, showing antimicrobial resistance profile, and the formation of biofilm aggregates, especially in nematode-infected cattle. Additionally, we propose that this increase in virulence may be linked to the synergistic pathogenicity of nematodes and bacteria.

## Materials and Methods

### Nematode isolation and identification

**Figure 1:**
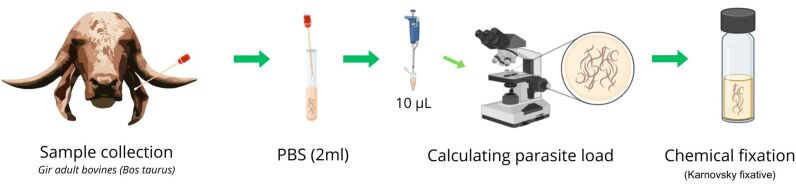
Nematodes were collected by inserting sterile swabs into the auditory canal of cattle, performing rotary movement, and immediately transferring them to sterile tubes containing 2 mL of PBS. To determine the parasite load, the tube contents were homogenized, and 500 µL aliquots were transferred to microtubes. The material was homogenized again, and 10 µL was immediately removed and analyzed in triplicate via bright-field light microscopy. The samples were fixed via immersion in Karnovsky solution.

Samples of auricular secretions were collected from twenty Gir adult bovines (*Bos taurus indicus*) aged between 2 and 17 years, ten randomly selected from the Piraí farm (22°38'32.8”S, 43°56'35.4”W) in the state of Rio de Janeiro and ten from the Castanhal farm (1°17'30.8”S, 48°08'25.0”W) in the state of Pará, Brazil. Both are dairy cattle farms. For auricular secretions, sterile swabs were inserted via rotary movements, immediately transferred to sterile tubes with 2 mL of PBS, and transported at 26–30 °C. Nematode presence was identified by analyzing 10 μL of the solution via light microscopy. For the determination of the parasite load, 1.5 mL samples were collected in sterile tubes and homogenized, and 500 µL was aliquoted into microtubes. The material was homogenized again, and 10 µL was removed immediately and mounted on slides, after which only adult worms were counted (Figure 1). All analyses were performed in triplicate, and the results are expressed as the means.

The isolated nematodes were fixed in 2.5% glutaraldehyde or 4% freshly prepared formaldehyde in 0.1 M cacodylate buffer (pH 7.2) (Lopes-Torres et al., 2013), and the species were morphologically identified via bright-field light microscopy, confocal microscopy (CM), scanning electron microscopy (SEM) and keys and original descriptions found in the current literature, Martins (1985), Sudhaus (2011), Asif et al. (2013) and Sudhaus (2023).

For confocal microscopy, samples were mounted in glycerin and analyzed with laser excitation at 488 nm for a fluorescein isothiocyanate (FITC) filter via a Leica TCS SP8 confocal microscope (Leica Microsystems, Germany) at CMABio (ESA/UEA) (Semprucci & Burattini, 2015). The isolated nematodes were identified and analyzed via confocal microscopy, which revealed autofluorescence with peak wavelengths at 495 nm excitation and 520 nm emission.

**Figure 2:**
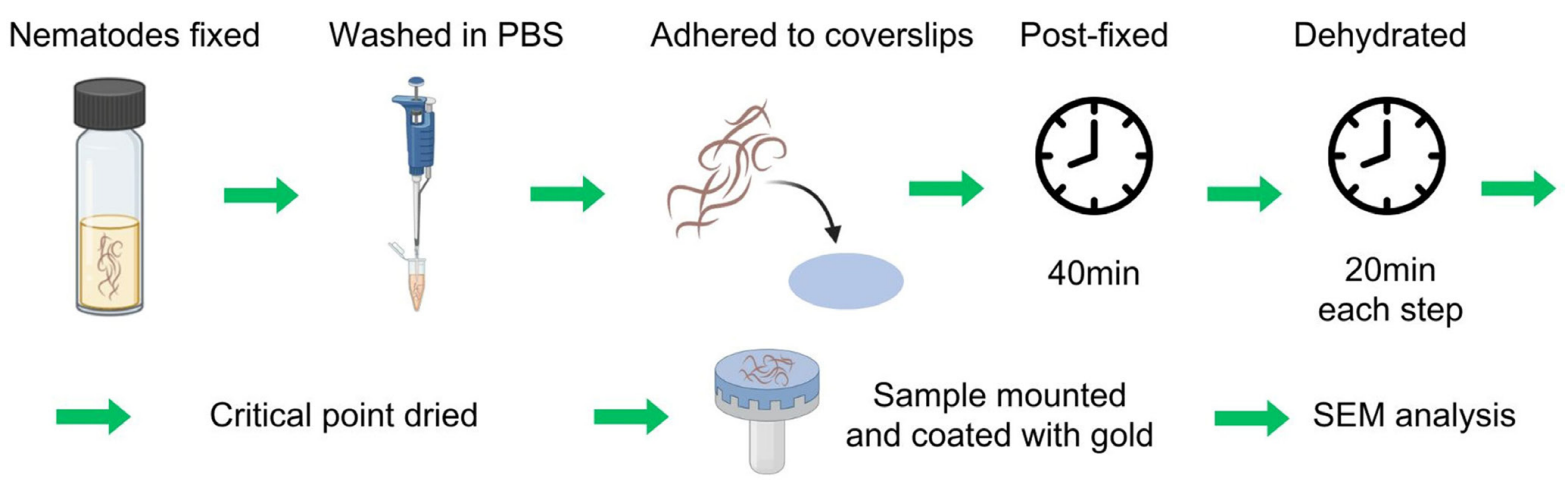
For ultrastructural analyses, the fixed worms were washed in PBS, adhered to coverslips with 1% gelatin, fixed in 1% OsO_4_ and 0.8% K_3_[Fe(CN)_6_], dehydrated in a graded ethanol series (30%-absolute), critical point dried in CO_2_, mounted on stubs, coated with gold, and examined via a scanning electron microscope.

For scanning electron microscopy (SEM) and field emission scanning electron microscopy (FESEM), fixed worms were washed in PBS, adhered to coverslips with 1% gelatin (Sigma-Porcine Skin), and postfixed in 1% OsO_4_ and 0.8% K_3_[Fe(CN)_6_] for 40 minutes. The samples were subsequently dehydrated in a graded ethanol series (30%-absolute) for 20 minutes at each step, critical point dried in CO_2_, mounted on stubs, coated with gold (15 nm for SEM or 5 nm for FESEM), and examined via different scanning electron microscopes: SEM FEI Quanta 250 (FEI, United States) (CENABIO-UFRJ), FESEM ZEISS Auriga 600 Compact (Zeiss, Germany) (IBRAG-UERJ), and FESEM Jeol JSM-7100F (Jeol, Japan) (NANOFAB-UERJ) (Figure 2).

### Bacterial studies

**Figure 3:**
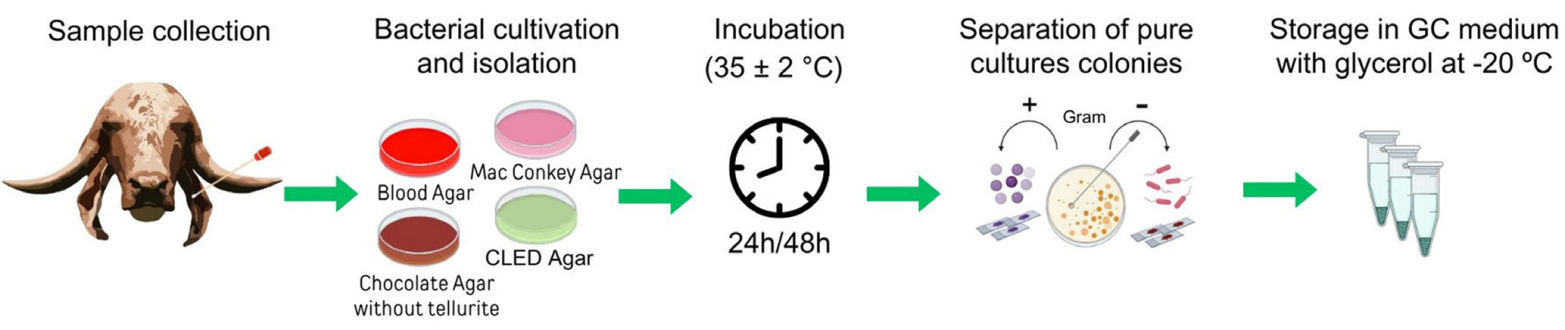
Bacterial collection and isolation were performed by inserting sterile swabs into the auditory canal of cattle and immediately transferring them to Stuart transport medium. Subsequently, various culture media plates were incubated at 35 ± 2 °C for 24 to 48 hours. Colonies that grew were selected for smear preparation and Gram staining to characterize their morphology and staining properties via bright-field light microscopy. Pure culture colonies were incubated in the same medium and stored in GC media supplemented with 20% glycerol at -20 °C.

The samples were collected from the external auditory canal with a sterile swab and immediately transferred to Stuart transport medium (CRAL, Brazil). The bacterial cultivation and isolation were performed in the following culture media plates: Cystine Lactose Electrolyte Deficient (CLED) agar (Kasvi, Brazil), Blood Agar (Laborclin, Brazil), Chocolate Agar without tellurite (Laborclin, Brazil), and MacConkey Agar (Kasvi, Brazil). The plates were incubated at 35 ± 2 °C for 24 hours or 48 hours for colony growth observation. The grown colonies were selected for smear analyzed by Gram staining, and their morphological and tinctorial characteristics were analyzed via light microscopy. For storage, pure culture colonies were incubated in the same medium at 35 ± 2 °C for 24 or 48 hours. The growth material obtained from each strain was stored in GC media (HiMedia laboratories, India) supplemented with 20% glycerol at -20 °C (Figure 3).

**Figure 4:**
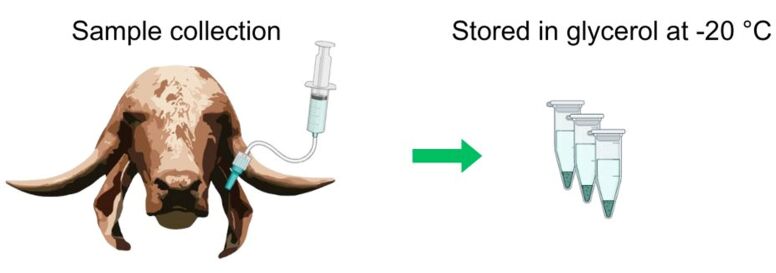
For mycoplasma collection, the ear canals were washed with 50 mL of sterile PBS via a syringe coupled with a hose. The samples were then stored in microtubes and transported in an isothermal box stored in glycerol at -20 °C.

To collect samples for mycoplasma analysis, the ear canal of the animal was rinsed with sterile phosphate-buffered saline (PBS, pH 7.2). The ear was washed with a syringe containing 50 mL of PBS coupled with a hose. The hose was gently introduced into the ear canal, and the wash was collected via the syringe itself. The samples were stored in identified microtubes and transported in an isothermal box stored in glycerol (1:2) at -20 °C (Figure 4).

The bacterial colonies were identified using Matrix-Assisted Laser Desorption/Ionization Time-of-Flight mass spectrometry (MALDI-TOF; BD^®^ Bruker MALDI Biotyper^®^ System, Bruker, Germany). Colonies were previously grown on Müeller‒Hinton agar (HiMedia Laboratories, India) for 18 hours at 35 ± 2 °C. Approximately ten colonies from each strain were collected, washed twice in 1 mL of sterile distilled water, resuspended in 1 mL of 70% ethanol, and centrifuged at 15,680 RCF for 2 minutes. The pellet was dried at 37 °C for 30 minutes, after which the cells were lysed in 100 μL of 35% formic acid in 50% acetonitrile. The mixture was centrifuged, and the supernatant was used for analysis. One microliter of each strain was applied to an air-dried metal plate, and 1 μL of the matrix (10 mg/mL α-cyano-4-hydroxycinnamic acid in 50% acetonitrile and 2.5% trifluoroacetic acid) was added. The microbial identification was performed via spectrum analysis in the database. The BioNumerics system, version 7.1, was used to analyze the protein spectra. The assay was performed in triplicate.

For the molecular identification of the mycoplasma, PCR tests were performed. An aliquot of 500 μL of each sample was subjected to DNA extraction using the phenol‒chloroform method, adapted from Sambrook & Russell (2006). Afterward, all samples and positive controls were quantified and evaluated for their degree of purity in a Biodrop Touch^®^ spectrophotometer (Biochrom, United States). PCR was performed as previously described (Table 1) by Van Kuppeveld et al. (1992) for the class Mollicutes; Chávez González et al. (1995) for *Mycoplasma bovis*; Buzinhani et al. (2007) for *Ureaplasma diversum*; Kobayashi et al. (1998) for *M. bovirhinis*, *Mycoplasma bovigenitalium*, and *Mycoplasma alkalescens*; Chalker et al. (2004) for *Mycoplasma arginini*; and Persson et al. (1999) for the *Mycoplasma mycoides* cluster (Table 1). The *M. bovis* Donetta, *M*. *agalactiae* PG2, *U*. *diversum* ATCC 49783, *M*. *bovirhinis* PG43, *M*. *bovigenitalium* PG11, *M*. *alkalescens* PG31, *M*. *arginini* G230, and *M*. *mycoides* subsp. *mycoides* LC Y Goat reference strains were used as positive controls. All reactions were performed in a Px2 thermocycler (Thermo Fisher, USA). The amplification products were applied to a 1.5% agarose gel submerged in Tris-Borate-EDTA (TBE) buffer and then subjected to an electrophoretic run at 75 volts (5 V/cm) for 60 minutes. Subsequently, the gel was stained with ethidium bromide (0.4 mg/mL) and visualized under ultraviolet light in a photodocumenter to observe the amplified products

**Table 1:** Description of primers, nucleotide sequence, size of the product obtained and respective bibliographic references for detecting classes Mollicutes, *Mycoplasma bovis*, *Ureaplasma diversum*, *M. bovirhinis*, *M. bovigenitalium* and *M. alkalescens*, *M. arginini*, and the *M. mycoides* cluster.

Primers*	**Sequence (5’ – 3’ direction)**	**Product**	**Reference**
GPO3	GGGAGCAAACAGGATTAGATACCCT	280pb	Van Kuppeveld et al. (1992)
MGSO	TGCACCATCTGTCACTCTGTTAACCTC		
MboF	CCTTTTAGATTGGGATAGCGGATG	360pb	Chávez-González et al. (1995)
MboR	CCGTCAAGGTAGCATCATTTCCTA		
UD3	AATGTCGGCTCGCTTATGAG	216pb	Buzinhani et al. (2007)
UD4	CCTGTCATATTGTTAACCTCCGC		
Mbg –F	CGTAGATGCCGCATGGCATTTACG	312pb	Kobayashi et al. (1998)
Mbg –R	CATTCAATATAGTGGCATTTCCTA		
Mbr-F	GCTGATAGAGAGGCTTATCG	316pb	
Mbr-R	ATTACTCGGGCAGTCTCC		
Mak-F	GCTGTTATAGGAAAGAAAACT	704pb	
Mak-R	AGAGTCCTCGACATGACTCG		
Myc 1F	CACCGCCCGTCACACCA	312pb	Chalker et al. (2004)
M.arginini	GTTGTATGACCTATTGTTGTC		
F-REAP	GAA ACG AAAGATAATACCGCATGTAG	785pb	Persson et al. (1999)
R-REAP	CCACTTGTGCGGGTCCCCGTC		

### Antimicrobial susceptibility test

Antimicrobial susceptibility testing was performed via the Kirby–Bauer disc diffusion method in Mueller–Hinton agar following BrCAST-EUCAST recommendations and guidelines (BrCAST-EUCAST, 2023). Fifteen commercial antimicrobials were used for the study (Cefar diagnóstica, Brazil), including beta-lactams (amoxicillin - 10 µg, ampicillin - 10 µg, cefoxitin - 30 µg, cefepime - 30 µg, cefazolin - 30 µg, cefuroxime - 30 µg, ceftriaxone - 30 µg, and meropenem - 10 µg), aminoglycosides (amikacin - 30 µg and gentamicin - 10 µg), macrolide (erythromyicin - 15 µg), fluoroquinolone (norfloxacin - 10 µg), amphenicol (chloramphenicol - 30 µg), sulfonamides (sulfamethoxazole + trimethoprim - 25 µg), and tetracycline (30 µg). Multidrug resistance (MDR) is defined as resistance to three or more classes of antimicrobials (Magiorakos et al., 2012). *Escherichia coli* ATCC 25922 and *Staphylococcus aureus* ATCC 2921 3 were used as reference strains.

### Biofilm formation

The biofilm formation test from bacterial colonies was performed in a 96-well polystyrene plate via crystal violet staining according to the method described by Stepanovic et al. (2007) with modifications. Colonies were diluted in tryptone-soy broth (HiMedia Laboratories, India), and the suspension concentration was adjusted to an optical density of 0.2 at a wavelength of 550 nm. A total of 200 µL of the bacterial suspension was added to the wells of a polystyrene plate and incubated at 37±1 °C for 48 h. Pure broth was used as a negative control. After incubation, the medium with the cells was removed, and the wells were washed six times with distilled water and dried. After drying, the plates were stained with 200 µL of 2% crystal violet solution for 5 min. The plate was washed with sterile water and dried at room temperature. The crystal violet bound to the biofilm was dissolved by the addition of 160 µL of 33% acetic acid. The absorbance was measured at 570 nm with a microplate reader on a spectrophotometer (Multiskan FC, Thermo Fisher, USA) at a wavelength of 570 nm. The results are expressed as optical density (OD) values, and the pattern of biofilm formation was considered the optical density value of the negative control (ODN) to establish the following criteria: nonadherent (−) OD ≤ ODN, weak (+) ODN < OD ≤ 2 × ODN, moderate (++) 2 × ODN < OD ≤ 4 × ODN, and strong (+++) 4 × ODN < OD (Umeda et al., 2017). Alternatively, some coverslips, with bacteria adhered either to form biofilms or not, were mounted on microscope slides and analyzed via an Olympus BX 53F (Olympus, Japan) microscope in bright-field mode. Images were captured via an Olympus Sc100 digital camera (Olympus, Japan).

## Results

### Effects of *Metarhabditis* infection on bacterial species associations

**Figure 5:**
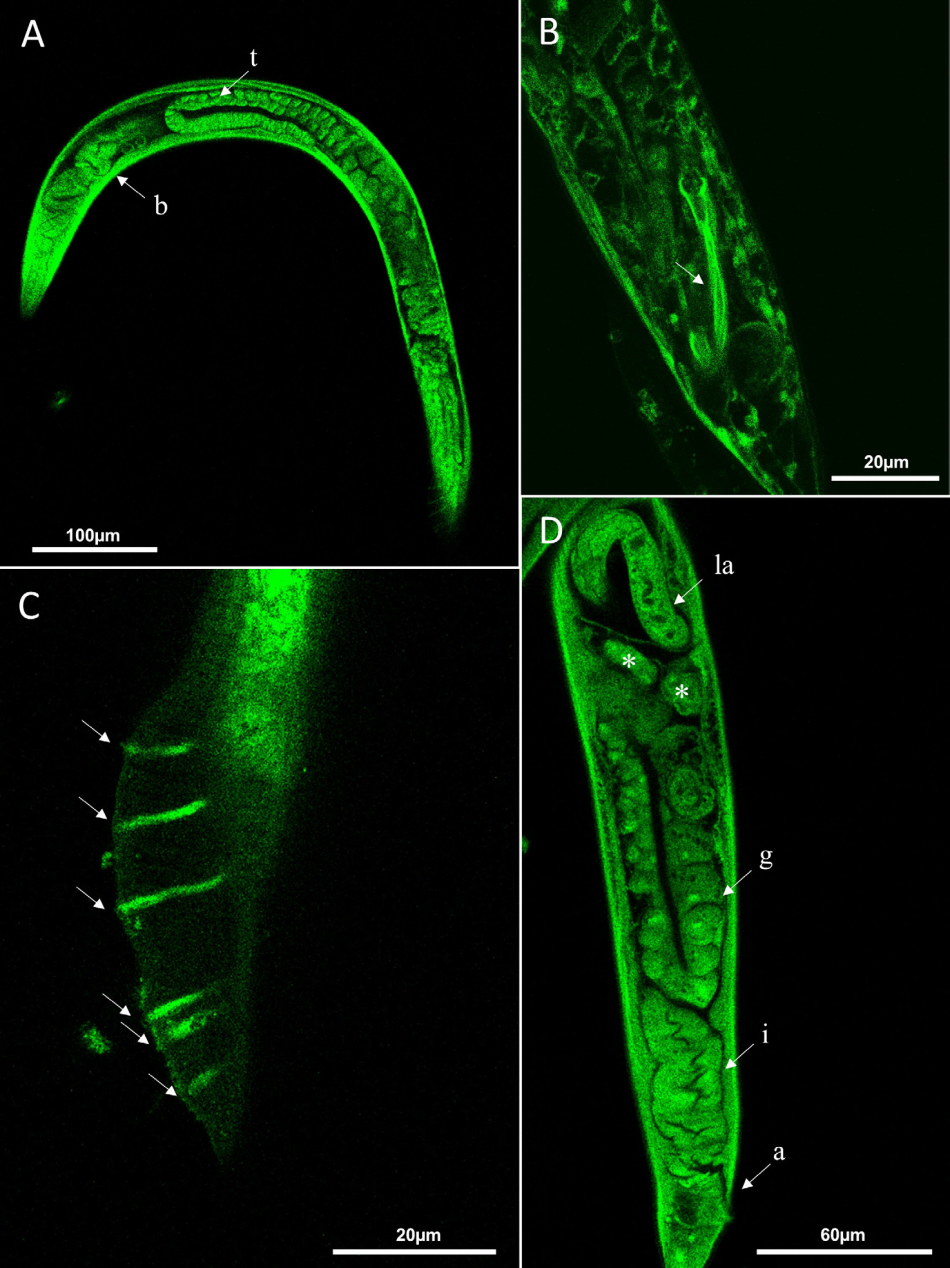
Confocal microscopy of *Metarhabditis* sp. **A-** General morphology of a male showing the esophageal bulb (b) and testis (t). **B-** Posterior region of the male, showing the spicule (arrow). **C-** Posterior end of the male showing the lateral view of the bursa and genital papillae (arrows). **D-** Posterior region of the female showing a larva (la), eggs (*), gonad (g), terminal region of the intestine (i), and anus aperture (a).

Nematodes *Metarhabditis freitasi* and *M*. *costai* were observed and identified in the ear canal secretions of 75% (15/20) of the total bovines examined: 60% (6/10) at Castanhal farm and 90% (9/10) at Piraí farm. The parasite load in 10 µL was 6.2 ± 5.7 (1-18) nematodes per animal at Piraí Farm and 134.5 ± 109.5 (26-259) per animal at Castanhal farm. In this study, we used bright field light microscopy, SEM and confocal microscopy to identify the nematodes *M*. *freitasi* and *M*. *costai* (Martins, 1985; Sudhaus, 2011). Specific structures were distinguishable, particularly at the posterior end of the male, such as the spicule, the number and organization of rays in the bursa, and the genital papillae. In females, it was possible to observe larvae inside the body, eggs in formation in the uterus, gonads, the terminal region of the intestine, and the anus aperture (Figure 5). In a careful analysis of the ear material collected from all animals, including both macroscopic and microscopic observations, no *Raillietia* specimens were detected in any of the samples.

**Figure 6:**
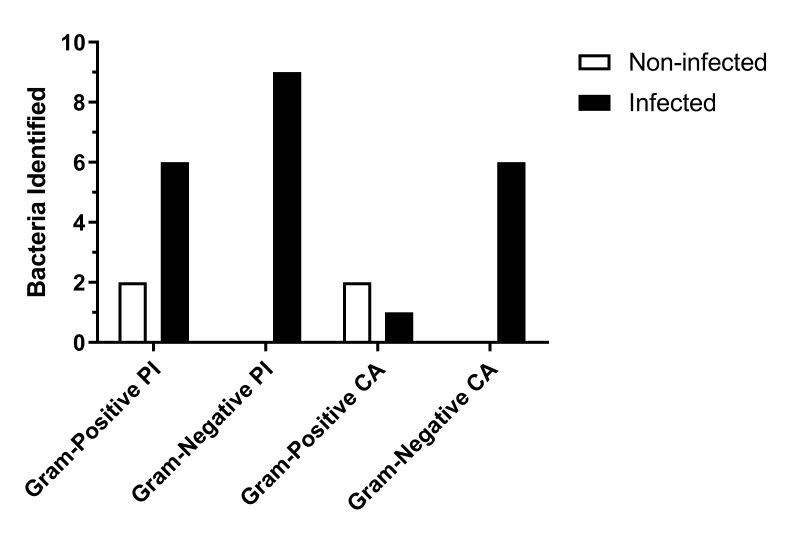
Number of Gram-positive and Gram-negative bacteria isolated from samples collected from the ear canal of bovines from Piraí (PI) and Castanhal (CA) farms. Gram-negative bacteria were isolated only from bovines infected with nematodes. Gram-positive bacteria were isolated from infected and noninfected cattle.

A bacterial study of the bovine auditory canal revealed the presence of Gram-negative bacteria in 50% (10/20) of these animals, Gram-positive bacteria in 35% (7/20), and mycoplasmas in 45% (9/20). In the group of nematode-infected cattle, we isolated a greater percentage of Gram-negative bacteria (GNB), which were identified in 66.7% (10/15) of these animals. Gram-positive bacteria (GPB) were identified in 33.3% (5/15) and *Mycoplasma* spp. in 45% (9/20) of the infected parasitized cattle. All GNB species and *Mycoplasma* spp. were identified only in parasitized bovines from both farms. Cattle without nematodes in the ear were identified with Gram-positive bacteria in 40% of the animals (2/5) (Table 2). For bacterial culture identification, we utilized the MALDI-TOF system, which revealed the diversity of Gram-negative bacteria (GNB) and Gram-positive bacteria (GPB) (Figure 6, Table 2).

**Table 2:** Characterization of bacterial species in the external auditory canal: MALDI-TOF mass spectrometry for Gram-negative and Gram-positive species, and PCR for *Mycoplasma* species in cattle from Piraí (white background) and Castanhal (gray background) farms.

**Animal**	**Condition**	**Gram-positive**	**Gram-negative**	***Mycoplasma*** *
1	Infected	-	*Escherichia coli*	*Mycoplasma bovis*, *Mycoplasma bovirhinis*
2	Infected	-	*Citrobacter koseri*, *E. coli*	-
3	Infected	*Staphylococcus cohnii*	*C. koseri*, *Serratia marcescens*, *E. coli*	*Mycoplasma* spp.
4	Infected	-	-	*Mycoplasma bovirhinis*
5	Infected	*Bacillus pumilus*	*S. marcescens*, *Acinetobacter lwoffi*	*Mycoplasma bovirhinis*
6	Non-infected	*Staphylococcus chromogens*, *Aerococcus viridans*, *Arthrobacter arilaitensis*	-	-
7	Infected	*Arthrobacter arilaitensis*, *S. chromogenes*	-	-
8	Infected	-	-	*Mycoplasma bovirhinis*
9	Infected	-	-	-
10	Infected	*Bacillus cereus*	*C. koseri*, *Oligella urethralis*	-
11	Infected	-	-	-
12	Infected	*S. chromogenes*	*C. koseri*	*Mycoplasma* spp.
13	Infected	-	*Morganella morgani*	*Mycoplasma bovis*
14	Infected	-	*M. morgani*, *S. marcescens*	*Mycoplasma bovis*
15	Infected	-	*S. marcescens*	*Mycoplasma* spp.
16	Non-infected	-	-	-
17	Non-infected	-	-	-
18	Non-infected	*Staphylococcus*	*-*	-
		*saprophyticus, Arthrobacter gandavensis*		
19	Non-infected	-	-	-
20	Infected	-	*M. morgani*	-

* Species detection was performed using PCR of the 5' UTR region.

In the fifteen infected animals, there was greater bacterial diversity, and the GPB species identified were *Staphylococcus chromogenes*, *Staphylococcus cohnii*, *Bacillus pumilus, Bacillus cereus*, and *Arthrobacter arilaitensis*. The GNBs identified in infected animals included *Serratia marcescens*, *Citrobacter koseri, Escherichia coli*, *Morganella morganii*, *Acinetobacter iwoffii*, and *Oligella urethralis*. In five non-parasitized animals, only Gram-positive species were identified: *Staphylococcus saprophyticus*, *Arthrobacter gandavensis*, *Staphylococcus chromogens*, *Aerococcus viridans*, and *Arthrobacter arilaitensis* (Table 2).

*Mycoplasma* species were identified in six cattle from the Piraí farm and four from the Castanhal farm, specifically in nematode-infected bovines (Table 2). PCR analysis detected *Mycoplasma bovis* in three animals, *M*. *bovirhinis* in four, and *Mycoplasma* sp. in three others. However, species-level confirmation through sequencing was not performed in this study. Our results, indicating the identification of pathogenic bacteria in nematode-infected bovines, suggest a relationship between the presence of *M*. *freitasi* and *M*. *costai* and increased bacterial diversity, including *Mycoplasma* spp.

### Nematode infection and antimicrobial resistance.

Among the twenty bacterial strains collected from infected and non-parasitized cattle, 80% (16/20) were resistant to at least one class of antimicrobial. Among the strains isolated from parasitized bovines, 93.3% (14/15) presented a resistance profile, whereas in non-parasitized animals, only 40% (2/5) presented a resistance profile. The studied strains displayed the following resistance profiles: 45% (9/20) to cefazolin; 40% (8/20) to amoxicillin and ampicillin; 35% (7/20) to cefuroxime; 20% (4/20) to cefoxitin, cefepime, chloramphenicol, and norfloxacin; 15% (3/20) to ceftriaxone; 10% to erythromycin (2/20); and 5% to tetracycline (1/20). Greater phenotypic resistance of the tested bacteria was observed for cefazolin (45% - 9/20) and ampicillin/amoxicillin (40% - 8/20) (Table 3).

**Table 3:** Frequency of phenotypical resistance in different bacterial strains isolated from the auditory canal of cattle with or without bovine otitis: susceptible samples (−), sensitivity test not recommended with such antimicrobial (NR), amikacin (AMI), amoxicillin (AMO), ampicillin (AMP), cefoxitin (CFO), cefepime (CFM), cefazolin (CFZ), cefuroxime (CRX), ceftriaxone (CRO), chloramphenicol (CLO), erythromycin (ERI), gentamicin (GEN), meropenem (MER), norfloxacin (NOR), sulfamethoxazole + trimethoprim (SUT), and tetracycline (TET). White background (infected) and gray background (noninfected).

**Species/strains (n=20)**	**Antimicrobials**														
	**AMI**	**AMO**	**AMP**	**CFO**	**CFM**	**CFZ**	**CRX**	**CRO**	**CLO**	**ERI**	**GEN**	**MER**	**NOR**	**SUT**	**TET**
*Escherichia coli*	_	50% (1/2)	50% (1/2)	_	_	_	_	_	50% (1/2)	NR	_	_	50% (1/2)	_	NR
10% (2/20)															
*Citrobacter koseri*	_	66.6% (2/3)	66.6% (2/3)	100% (3/3)	66.6% (2/3)	100% (3/3)	100% (3/3)	66.6% (2/3)	33.3% (1/3)	NR	_	_	33.3% (1/3)	_	NR
15% (3/20)															
*Morganella morgani*	_	66.6% (2/3)	66.6% (2/3)	33.3% (1/3)	33.3% (1/3)	66.6% (2/3)	33.3% (1/3)	33.3% (1/3)	33.3% (1/3)	NR	_	_	_	_	NR
15% (3/20)															
*Serratia marcescens*	_	50% (2/4)	50% (2/4)	-	_	100% (4/4)	75% (3/4)	_	_	NR	_	_	_	_	NR
20% (4/20)															
*Staphylococcus cohni*	_	_	_	_	_	_	_	_	_	_	_	_	_	_	_
5% (1/20)															
*Staphylococcus chromogenes*	_	50% (1/2)	50% (1/2)	_	_	_	_	_	50% (1/2)	100% (2/2)	_	_	_	_	50% (1/2)
10% (2/20)															
*Staphylococcus chromogenes*	_	_	_	_	_	_	_	_	_	_	_	_	_	_	_
5% (1/20)															
*Staphylococcus saprophyticus*	_	_	_	_	_	_	_	_	_	_	_	_	_	_	_
5% (1/20)															
*Bacilus pumilus*	_	_	_	_	_	_	_	_	_	_	_	_	_	_	_
5% (1/20)															
*Arthrobacter arilaitensis*	_	_	_	_	_	_	_	_	_	_	_	_	100% (1/1)	_	_
5% (1/20)															
*Arthrobacter gandavensis*	_	_	_	_	_	_	_	_	_	_	_	_	100% (1/1)	_	_
5% (1/20)															

A multidrug resistance (MDR) profile was identified in some of the bacterial strains studied. The *Escherichia coli* and *Citrobacter koseri* strains exhibited resistance to the amphenicol, fluoroquinolone, and beta-lactam classes, with 100% (3/3) resistance to first- and second-generation cephalosporins observed in the *Citrobacter koseri* strains. Additionally, another significant pattern of MDR was observed in the *C. koseri* and *Morganella morganii* strains, which were resistant to ceftriaxone and cefepime and to third- and fourth-generation cephalosporins, respectively, indicating an extended-spectrum β-lactamase (ESBL) profile.

When comparing the same species, *Staphylococcus chromogenes*, isolated from infected and noninfected cattle, different profiles were observed in the antimicrobial sensitivity tests. Strains isolated from parasitized animals were resistant to four classes of antimicrobials (beta-lactams, amphenicol, macrolides, and tetracycline), whereas strains isolated from non-parasitized bovines were susceptible to all antimicrobials tested. Additionally, the strains of *Staphylococcus saprophyticus* and *Bacillus pumilus*, which were isolated from non-parasitized animals, were also susceptible to all classes of antimicrobials tested (Table 3). On the other hand, all tested strains demonstrated sensitivity to amikacin, gentamicin, meropenem, and sulfamethoxazole + trimethoprim.

### Nematode and biofilm formation

**Figure 7:**
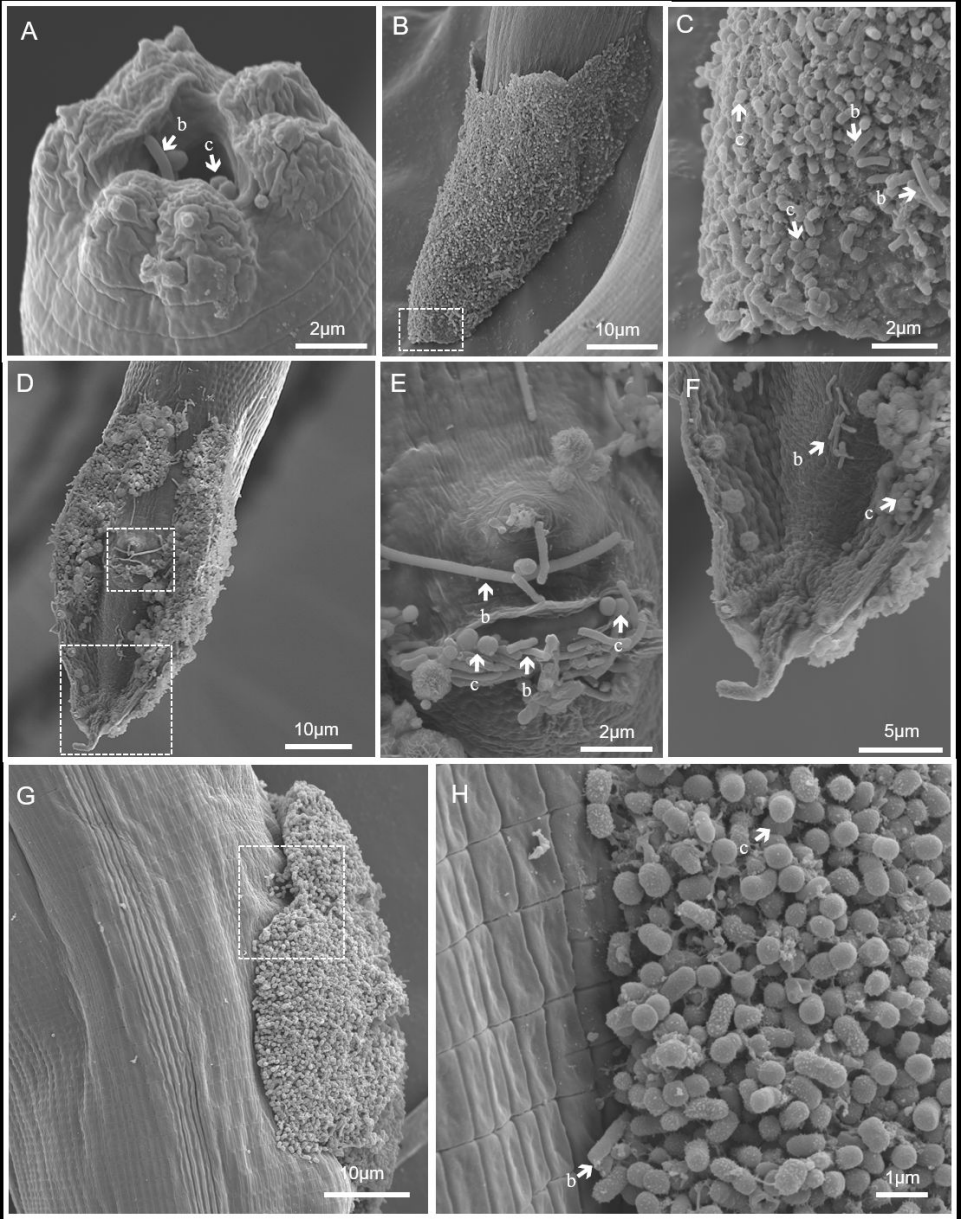
Scanning electron microscopy of bacteria adhered to the cuticle of *Metarhabditis* sp. **A-** Anterior end showing bacilli (b) and cocci (c) adhering to the oral opening. **B-** Dorsal view of the posterior region of a male completely covered by the biofilm-like structure. **C**-Details of the biofilm-like structure showing bacilli (b) and cocci (c) forming a dense packing of bacteria on the nematode cuticle. **D-** Ventral view of the male posterior region showing a biofilm-like structure. **E-** Details of cloacal opening with bacilli (b) and cocci (c). **F-** Tip of the posterior end showing adhered bacteria. **G-** Lateral view of the middle region of the female, showing the biofilm-like covering of the opening of the vulva. **H-** Details of the biofilm-like structure showing bacilli (b) and cocci (c) forming a dense packing of bacteria on the opening region of the nematode vulva.

Scanning electron microscopy of *Metarhabditis* nematodes recovered from the external auditory canal of bovines revealed a significant presence of bacteria adhered to the cuticle surface, indicating biofilm formation (Figure 7). At the anterior end (Figure 7a), bacilli and cocci were identified in the nematode oral opening. Notably, in male nematodes, biofilm bacterial aggregates were predominantly observed in the posterior region, covering the dorsal and ventral faces of the bursal structure (Figure 7b-d). The biofilm aggregate was concentrated on the ventral surface, at the posterior end edges (Figure 7d), and at the cloacal opening (Figures 7e and f). In females, the biofilm structure covered the vulva opening (Figures 7g and h), and an ultrastructural examination of these aggregates revealed diverse morphologies of bacteria (bacilli and cocci) adhering to each other, forming a dense material on the nematode cuticle (Figures 7 and h).

**Figure 8:**
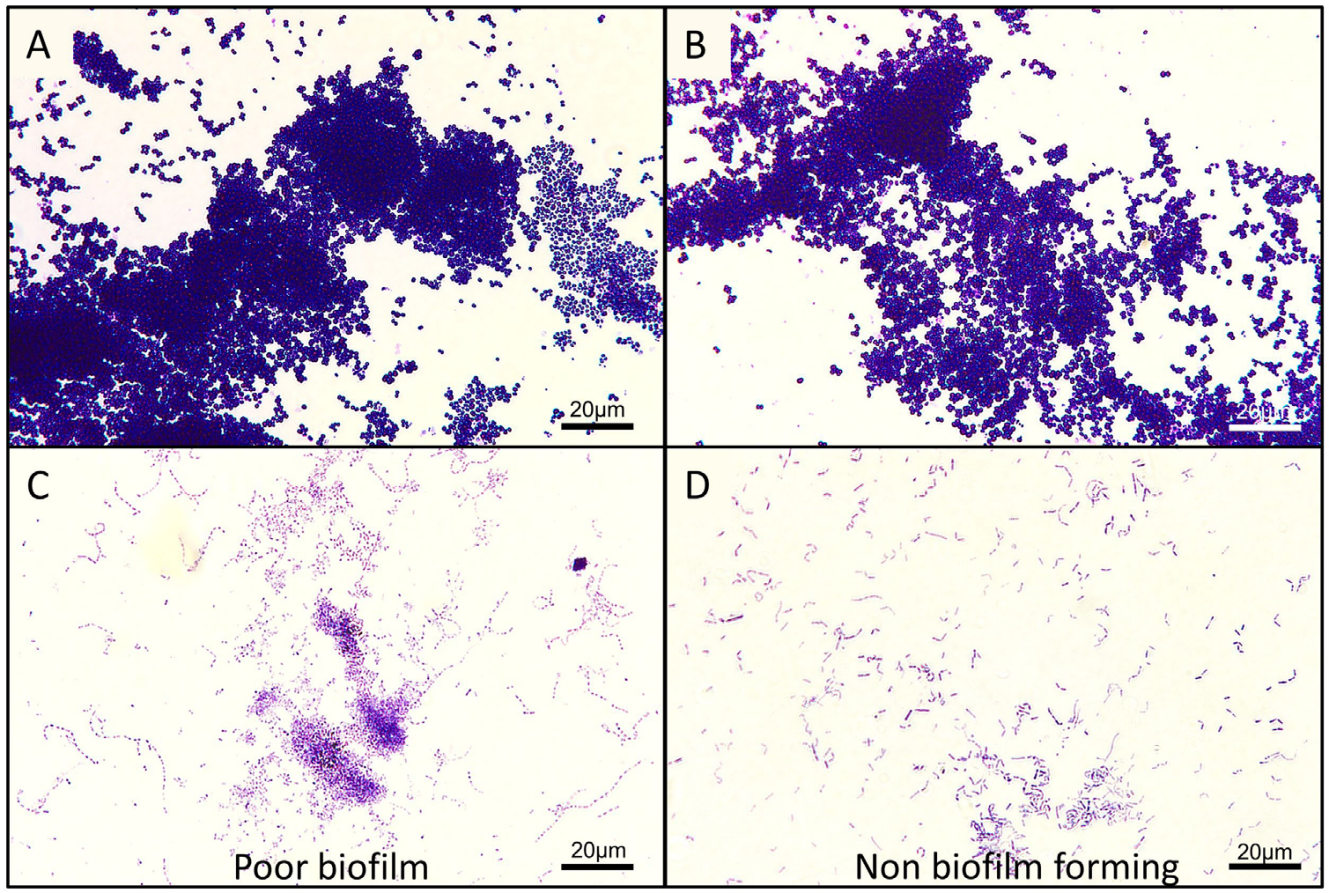
Light microscopy (bright-field) images of biofilm formation experiments. **A** and **B-** *Staphylococcus chromogenes* (strain 7) forming a moderate biofilm. **C-** *Escherichia coli* (strain 3 - CLED 1) forming a poor biofilm. **D-** *Bacillus pumilus* (strain 6 - AS3) showing no biofilm formation.

Owing to this discovery, we conducted biofilm formation assays on thirteen strains associated with the issue of antimicrobial resistance. Our analysis revealed that 84.6% (11/13) of the strains formed biofilms, whereas 15.4% (2/13) did not form biofilms (Table 4). In our quantitative determination experiments involving the eleven biofilm-forming samples, 72.7% (8/11) displayed a moderate ability to form biofilms (Figure 8a and b), and 27.3% (3/11) exhibited a poor capacity for biofilm formation (Table 4). Notably, among the strains capable of forming biofilms, 81.8% (9/11) were isolated from nematode-infected cattle, and 18.2% (2/11) were from non-parasitized animals. The species *Staphylococcus chromogenes*, known for its natural biofilm-forming ability, was identified, and *Arthrobacter gandavensis* was associated with human gum infections as well as mastitis and uterine diseases in cows.

**Table 4:** Biofilm formation ability of bacterial strains isolated from the auditory canal of parasitized and non-parasitized cattle. White background (Piraí farm) and gray background (Castanhal farm).

**Animal**	**Condition**	**Species/strains**	**Biofilm formation**
3	Infected	*Escherichia coli*	Poor biofilm
3	Infected	*Serratia marcescens*	Moderate biofilm former
3	Infected	*Staphylococcus cohni*	Moderate biofilm former
5	Infected	*Serratia marcescens*	Moderate biofilm former
6	Non-infected	*Arthrobacter arilaitensis*	Non biofilm forming
6	Non-infected	*Bacilus pumilus*	Non biofilm forming
6	Non-infected	*Staphylococcus chromogenes*	Moderate biofilm former
7	Infected	*Staphylococcus chromogenes*	Moderate biofilm former
12	Infected	*Staphylococcus chromogenes*	Moderate biofilm former
14	Infected	*Morganella morgani*	Moderate biofilm former
14	Infected	*Serratia marcescens*	Moderate biofilm former
18	Non-infected	*Arthrobacter gandavensis*	Poor biofilm
20	Infected	*Morganella morgani*	Moderate biofilm former

All strains (9/9) isolated from parasitized cattle presented notable potential for biofilm formation, as detailed in Table 4. Strikingly, among these strains, 88.8% (8/9) also demonstrated resistance to antimicrobials. This robust correlation between biofilm-forming potential and antimicrobial resistance further underscores the compelling hypothesis that parasitism significantly enhances bacterial virulence (Table 4, Figure 8).

## Discussion

Martins (1985) first reported the *Rhabditis* genus in association with Gir cattle in Brazil, including the first taxonomic description of *Rhabditis* (*Rhabditis*) *freitasi* Martins, 1985 and *Rhabditis* (*Rhabditis*) *costai* Martins, 1985. Posteriorly, both species were reallocated under the genus *Metarhabditis* (Sudhaus, 2011), and Caracciolo et al. (2024) utilized integrative taxonomy to study *Metarhabditis* species associated with Gir cattle. The present study reports new occurrences of this disease caused by nematodes *M*. *freitasi* and *M*. *costai* in two different states from distant regions of Brazil (southeast and north), highlighting the continental dimensions of the country. On both farms, the parasitized animals appeared asymptomatic, exhibiting normal auricular secretions without any visible alterations, such as purulent consistency or unpleasant odor. According to Leite et al. (2013) and Santos et al. (2024), the asymptomatic characteristics are considered to indicate subclinical otitis. However, the farmers at the Piraí farm reported that six months prior to the sample collection for this study, they had lost two cattle to otitis, which had only treated for the most severe symptoms.

On the farm in Castanhal, there was also a report of the death of a bovine six months before collection in this study, with advanced otitis. On both farms studied, the use of ivermectin “pour on” and washing of the ear canal with disinfectant solutions was reported, both as a preventative measure and as a treatment. The use of ivermectin has already been described as having low efficacy (Msolla et al., 1985; Verocai et al., 2009; Leite et al., 2013).

The physiopathology of bovine otitis is intricately shaped by the dynamic interplay of direct nematode–host tissue interactions and the existing ear microbiota. Our study revealed prevalent infection with *Metarhabditis* nematodes in the majority of bovines, with ear canal contents collected randomly. This finding suggests a possible association between the presence of the nematode and an increase in pathogenic bacterial diversity, indicating antimicrobial resistance and biofilm formation on the surface of the helminths.

Parasitic bovine otitis, associated with nematodes (Rhabditidae), has been registered in various countries, particularly in connection with dairy cattle (Msolla et al., 1993; Ushewokunze-Obatolu et al., 1999; Cardona et al., 2012). In Brazil, the prevalence of parasitic bovine otitis associated with *Metarhabditis* species has been reported extensively, with occurrence rates reaching 60–93% in Minas Gerais and 53% in Goiás (Duarte et al., 2001). Our current study, which was conducted in Rio de Janeiro and Pará states revealed prevalence rates of 90% (9/10) and 70% (7/10), respectively, in randomly selected animals. Previous research has highlighted a significantly greater prevalence of parasitic otitis in mature adult cows with horns (Duarte et al., 2001).

Our findings show a predominance of enterobacteria (GNB) in nematode-infected cattle, suggesting possible contamination from the soil to the ear canal. Similar observations were made by Yap & Gause (2018) in their study investigating the relationship between the murine intestinal nematode parasite *Heligmosomoides polygyrus*, which exacerbates the inflammatory process, thereby promoting the pathogenesis of *Salmonella* sp. and *Citrobacter* sp. In a related context, Schachter et al. (2020) demonstrated that *T*. *muris* infection increased the diversity and abundance of bacterial species in the intestinal microbiota when compared with uninfected mice. Intestinal and entomopathogenic nematodes live in intimate contact with host tissues, and their excretion/secretion products can interfere with host immunology and the microbiota. Changes in microbiome composition during nematode infection, which shift the abundance of bacterial groups, have been linked to the immunoregulatory potential of nematodes (Rausch et al., 2018; Jones et al., 2022). The inflammatory process in the cattle ear canal and the potential modulation of the immune system due to helminth parasitism can appear to be important factors contributing to the persistence of distinct bacterial groups when compared with those in the native microbiota.

The evaluation of pathogenic bacterial groups from the ears of nematode-infected cattle revealed important findings. Studies on *E*. *coli* in cattle otitis are limited. However, this species has been associated with otitis externa in dogs and cats (Nardoni et al., 2014). The identification of bacterial species such as *E*. *coli* in cattle raises concerns about the potential contamination of milk and dairy products, especially given the documented cases of antimicrobial resistance (Garbaj et al., 2016). *Citrobacter koseri*, known for its association with meningitis in calves, diarrhea, and septicemia (Fecteau et al., 2009), was identified. Additionally, *Serratia marcescens*, which was isolated for the first time from the ear canal of cattle, has been associated with bovine mastitis (Bi et al., 2016). We also identified *Arthrobacter arilaitensis*, which was previously isolated from cow milk and is associated with bovine mastitis and uterine infections (Braga et al., 2018; Verdier-Metz et al., 2012). Furthermore, *Staphylococcus chromogenes,* known for its strong biofilm-forming potential, was isolated from both farms, highlighting its possible significance in increasing pathogenicity, particularly through biofilm formation in association with nematodes (Vanderhaeghen et al., 2014; Israel et al., 2018). The identification of these pathogenic species underscores the impacts and risks of nematode–bacteria associations in dairy cattle otitis.

We were able to associate the presence of *Mycoplasma* spp. with nematode-infected animals from both farms. Santos et al. (2009) described several species of mycoplasmas in the ear canals of cattle, including *M. bovis* and *M*. *bovirhinis*. *M. bovis* is the predominant pathogen isolated from bovine ears and is associated with otitis, followed by *M. bovirhinis* and *M. alkalescens* (Francoz et al., 2004; Lamm et al. 2004). Previous studies have highlighted the potential role of mites (*Raillietia* spp.) in transmitting *Mycoplasma* (Santos et al., 2012). Despite the detection of *M*. *bovis* in the PCR of the 5' UTR region, further studies using additional genetic regions and/or sequencing are necessary to confirm the presence of this species in the auditory canal of cattle. However, this study provides the first evidence of nematode parasitism involving *M*. *bovirhinis*, suggesting that *Metarhabditis* spp. could act as a carrier for *Mycoplasma* and other microorganisms. These findings contribute to the understanding of the possible association of parasites as carriers with the transmission of bacteria.

Different species of *Metarhabditis* and *Rhabditids* have been isolated from soil directly or from soil insects in various countries of Asia, Europe, North America, and South America (Carta & Osbrink, 2005; Bhat et al., 2020; Donhauser et al., 2023). Bacterial-feeding nematodes exhibit a wide dietary range, showing a preference for bacteria that support their growth and reproduction (Lewenza et al., 2014; Liu et al., 2017). This selective feeding behavior, also observed in *Caenorhabditis elegans*, affects metabolic processes, longevity, and egg production (Venette & Ferris, 1998; Komura et al., 2022). These nematodes preferentially feed on GNB over GPB, likely due to the easier digestion of GNB (Zhou et al., 2023). This finding suggests that the high prevalence of GNB found in the ears of nematode-infected cattle may be associated with the presence of these bacterial groups in the digestive system of the nematodes, which may have been transported by mechanical vectors from the soil to the bovine auditory canal.

In the treatment of parasitic otitis in dairy cattle breeders, various strategies have been applied to reduce infection and alleviate animal pain and suffering, but no established protocol has been developed in Brazil or other countries (Santos et al., 2024). In this study, we observed a notable pattern of resistance, with 80% of strains showing resistance to commonly used antimicrobial classes, particularly those isolated from nematode-parasitized cattle. Strains from parasitized cattle presented multidrug resistance profiles, indicating that the environmental conditions induced by nematodes may contribute to increased expression of virulence factors, potentially leading to increased antibiotic resistance (Rausch et al., 2018). These findings are consistent with those of Hinthong et al. (2017), who reported high multidrug resistance to antimicrobials in *E*. *coli* isolates from bovine mastitis. The observation of multidrug resistance in *E*. *coli* is particularly concerning because of its versatile ability to acquire and transmit resistance genes (Souza et al., 2023).

The observed resistance to beta-lactams in the evaluated strains is noteworthy, given the widespread use of these broad-spectrum antibiotics in treating bovine mastitis, prophylaxis, and growth promotion, often in sub doses over extended periods (Sharun et al., 2021). This practice contributes to the emergence of resistance, underscoring the significance of this finding.

A high frequency of GNB, including biofilm-producing species such as *Serratia marcescens*, has been reported in nematode-infected cattle (Stocco et al., 2017). Our studies revealed that Enterobacteriaceae and some species of *Staphylococcus*, especially *S*. *chromogenes*, have moderate biofilm-forming abilities, as observed by Israel et al. (2018) in mastitis-affected milk samples. Biofilm formation is crucial for antimicrobial resistance and facilitates bacterial dispersion to other sites, increasing their invasive potential (Gebreyohannes et al., 2019). Scanning electron microscopy (SEM) stands out as an optimal tool for visualizing biofilm formation, particularly when high-resolution images are essential for accurately describing the morphology and organization of microorganisms (Relucenti et al., 2021). Our SEM images of microbial agglomerations attached to *Metarhabditis* nematodes suggest that these parasites may act as vectors of pathogenic bacteria on the cuticle or in the gut, as observed in previous studies with *C*. *elegans*, which revealed that biofilm-forming bacteria can survive internally within this nematode (Smolentseva et al., 2017). Adult worms or larvae of *Metarhabditis* nematodes can migrate through ear tissues with bacteria adhering to their cuticle, leading to bacterial translocation to different tissues in areas close to the brain. This could provide an explanation for the documented nervous system disorders associated with severe bovine parasitic otitis (Duarte & Hamdan, 2004).

The multiresistance drug profile observed in bacteria isolated from cattle with otitis raises concerns regarding potential environmental and human contamination through meat, milk, and derivatives, as well as contact with the animals. This finding reinforces the need for further One Health studies and discussions on the rational and controlled use of treatment strategies in livestock (Medina-Pizzali et al., 2021). All the strains tested demonstrated susceptibility to amikacin, meropenem, gentamicin, and sulfamethoxazole + trimethoprim, which is crucial for identifying potentially more effective antimicrobials for treating coinfections in bovine otitis. Strategically and carefully using these antimicrobials can be key in minimizing the emergence of antimicrobial resistance (Mshana et al., 2021).

Our study highlights gaps in the understanding of the transmission, development, and treatment of parasitic otitis, particularly the intricate relationship between nematodes and ear canal microbiota. Severe bovine otitis may result from a combination of macroparasites (*Raillietia* spp. and *Metarhabditis* spp.) and pathogenic bacteria, presenting a complex challenge that requires further exploration. Treatment is complicated by increased bacterial virulence, antimicrobial resistance, multidrug resistance, and biofilm formation potential. The increase in bacterial virulence in parasitic coinfections serves as a critical One Health warning, urging additional studies to provide new information about the parasite-host-microbiota relationship and opening new strategies for parasitic control and treatment. Further studies, including *in vivo* assays, are necessary to develop efficient treatment protocols. However, our results provide important guidance for researchers and dairy cattle breeders in developing and improving new protocols.
